# Relationship of Event-Related Potentials to the Vigilance Decrement

**DOI:** 10.3389/fpsyg.2018.00237

**Published:** 2018-03-06

**Authors:** Ashley Haubert, Matt Walsh, Rachel Boyd, Megan Morris, Megan Wiedbusch, Mike Krusmark, Glenn Gunzelmann

**Affiliations:** ^1^Sensor Systems Division, Human Factors Group, University Research Institute, Dayton, OH, United States; ^2^RAND Corporation, Santa Monica, CA, United States; ^3^Georgia Institute of Technology, Atlanta, GA, United States; ^4^Ball Aerospace & Technologies, Fairborn, OH, United States; ^5^Oak Ridge Institute for Science and Education, Oak Ridge, TN, United States; ^6^L3 Technologies, New York, NY, United States; ^7^Air Force Research Laboratory, Dayton, OH, United States

**Keywords:** ERP (event related potentials), vigilance decrement, N1 amplitudes, P3b, resource-control theory, resource-depletion, mind-wandering, modeling

## Abstract

Cognitive fatigue emerges in wide-ranging tasks and domains, but traditional vigilance tasks provide a well-studied context in which to explore the mechanisms underlying it. Though a variety of experimental methodologies have been used to investigate cognitive fatigue in vigilance, relatively little research has utilized electroencephalography (EEG), specifically event-related potentials (ERPs), to explore the nature of cognitive fatigue, also known as the vigilance decrement. Moreover, much of the research that has been done on vigilance and ERPs uses non-traditional vigilance paradigms, limiting generalizability to the established body of behavioral results and corresponding theories. In this study, we address concerns with prior research by (1) investigating the vigilance decrement using a well-established visual vigilance task, (2) utilizing a task designed to attenuate possible confounding ERP components present within a vigilance paradigm, and (3) informing our interpretations with recent findings from ERP research. We averaged data across electrodes located over the frontal, central, and parietal scalp. Then, we generated waveforms locked to the onset of critical low-frequency or non-critical high-frequency events during a 40 min task that was segregated into time blocks for data analysis. There were three primary findings from the analyses of these data. First, mean amplitude of N1 was greater during later blocks for both low-frequency and high-frequency events, a contradictory finding compared to past visual vigilance studies that is further discussed with respect to current interpretations of the N1 in visual attention tasks. Second, P3b mean amplitude following low-frequency events was reduced during later blocks, with a later onset latency. Third and finally, the decrease in P3b amplitude correlated with individual differences in the magnitude of the vigilance decrement, assessed using *d*′. The results provide evidence for degradations of cognitive processing efficiency brought on by extended time on task, leading to delayed processing and decreased discriminability of critical stimuli from non-critical stimuli. These conclusions are discussed in the context of the vigilance decrement and corresponding theoretical accounts.

## Introduction

Cognitive fatigue encompasses a variety of phenomena related to decrements in cognitive performance associated with time-on-task (Ackerman and Kanfer, 2009). One of the most well-studied manifestations of cognitive fatigue is the vigilance decrement, which is defined as a decreased probability of detecting rare critical events in streams of stimuli with increased time-on-task (Mackworth, 1948). The vigilance decrement has been investigated in a myriad of settings, which has led to the conclusion that “mental fatigue” and sustained attention are conceptually similar, if not identical (Oken et al., 2006). “Mental fatigue” can be understood as a progressive decrease in vigilance that is exacerbated by time spent on a tedious but demanding task (Charbonnier et al., 2016).

Understanding the *vigilance decrement* is not only of scientific interest, but is also of practical importance. The vigilance decrement occurs across a range of work settings including air traffic control (Brookings et al., 1996), power transmission control operation (Small et al., 2014), nuclear power plant operation (Reinerman-Jones et al., 2016), closed circuit television security monitoring (Näsholm et al., 2014), and airport baggage screening (Wolfe et al., 2005). The decrement has been shown to occur even when people are allowed to confirm, and potentially correct, incorrect responses (Van Wert et al., 2009). The defining feature across these examples is that the vigilance decrement is a consequence of performing attention-demanding tasks over time spans ranging from tens of minutes to hours (e.g., See et al., 1995).

Research has given rise to two broad classes of accounts for understanding the vigilance decrement: *overload* accounts and *underload* accounts. Overload accounts propose that the decrement occurs because the effort required in vigilance tasks depletes limited information processing resources that are crucial for performing the task, leading to reduced detection of critical events (Helton and Warm, 2008; Thomson et al., 2015). The resource-depletion hypothesis, an exemplary overload account, claims that increases in task demands or time-on-task consumes resources faster than they can be replenished, which produces a vigilance decrement (Warm et al., 1996, 1998; Grier et al., 2003; Small et al., 2014).

In contrast to overload accounts, underload accounts claim that the lack of stimulation, or the monotony of vigilance tasks causes attention to shift from the external task. Performance then declines, due to automaticity of task performance or as a result of distraction by unrelated thoughts (Helton and Warm, 2008; Thomson et al., 2015). From this perspective, attentional lapses are a result of perceptual decoupling caused by the monotonous, non-engaging nature of the task (Robertson et al., 1997; Manly et al., 1999). Most underload accounts address what causes the initial decrease in performance (understimulation of the task), but few have tried to explain where attention is shifted. The mind-wandering hypothesis, an exemplary underload account, proposes that attention shifts to self-generated unrelated thoughts, leading to performance decrements (Manly et al., 1999; Smallwood and Schooler, 2006; Smallwood, 2013; Axelrod et al., 2015).

Another recent account, the resource-control theory, seeks to reconcile overload and underload accounts (Thomson et al., 2015). The resource-control theory holds that executive control works to prevent mind-wandering, but requires cognitive effort. This effort leads to decrements in executive function that manifest as mind-wandering, which diverts attentional resources from the task to off-task cognitive activities. Consistent with overload accounts, crucial resources are taxed by the effort required to remain focused on the task (executive control). Consistent with underload accounts, mind-wandering undermines performance on the primary task through distraction. The authors argue that this theory uniquely accounts for the complete range of findings that have been reported in the vigilance literature.

Recently, we have proposed a computational model to account for the vigilance decrement (Veksler and Gunzelmann, 2017). The computational model is generally consistent with resource-control theory. In the model, vigilance tasks tax central cognitive resources associated with engaging in goal-directed cognitive processing. When those resources are depleted, goal-directed processing is disrupted by *microlapses* – brief gaps in cognitive processing that disrupt performance. *Microlapses* are distinct from *mind-wandering* in that mind-wandering is a redirection of cognitive processing, but both have the same effect on vigilance performance – disruptions in goal-directed cognition needed to detect critical signals.

Despite the assortment of factors that influence the vigilance decrement, theoretical debates about the underlying mechanisms have not been resolved. Many factors have been shown to influence the timing and magnitude of the vigilance decrement (Gartenberg et al., 2014), including signal duration (Baker, 1963), source complexity (Teo and Szalma, 2011), and use of declarative memory in the task (Desmond et al., 2001). Stimulus event rate (Lanzetta et al., 1987; Parasuraman and Mouloua, 1987) has been shown to impact the size of the decrement in modified vigilance tasks such as an n-back working memory-updating task (Borragán et al., 2017). In addition, the timing of the vigilance decrement varies depending on the task demands. Performance declines typically occur within the first 20–35 min of a task, with half of the decrement seen in the first 15 min (Teichner, 1974). The onset of the decline may come sooner in more challenging tasks (See et al., 1995). These findings, though highly replicable, do not definitively speak to core mechanisms.

Researchers have turned to physical measures to elucidate debates regarding the vigilance decrement. Some vigilance studies have used a transcranial doppler, an ultrasound device that monitors blood flow via arteries in the brain, to measure changes in cerebral blood flow velocity with time-on-task. These studies reveal that overall blood flow decreases with time-on-task (Hitchcock et al., 2003; Matthews et al., 2010; Shaw et al., 2013), with these effects primarily lateralized to the right hemisphere (Warm et al., 2009; Matthews et al., 2010). Functional magnetic resonance imaging (fMRI), a neuroimaging technique that detects changes in blood oxygenation, also corroborates that sustained attention primarily affects activation in regions in the right hemisphere (Lewin et al., 1996). Still, there is little known about the underlying mechanisms of the vigilance decrement (Boksem et al., 2005), and no decisive resolution to the theoretical debates have emerged.

One limitation of transcranial Doppler and fMRI is a lack of temporal precision needed to fully identify what aspects of processing are affected by the vigilance decrement. Electroencephalography (EEG), however, provides a near-continuous measure of cognitive processing with millisecond resolution by measuring neuro-electrical activity at the scalp that originates from neurons in the brain. EEG signals relate to sensory, motor, and cognitive processes of interest. Some portion of the EEG signal may be evoked by experiment events, such as stimulus presentation and response execution, giving rise to event-related potentials (ERPs). As such, the EEG methodology can provide nuanced understanding in which stages of cognitive processing – stimulus encoding, categorization, and/or response selection – are impacted by the vigilance decrement.

Existing EEG studies, though informative with respect to the vigilance decrement, provide an incomplete, and sometimes inconsistent, account. The majority of studies that analyzed time-on-task effects did not involve traditional vigilance paradigms. Indeed, in most cases, the paradigms used were designed to study some other phenomenon, and time-on-task effects were of secondary interest. This limits the applicability of ERP results to theories of the vigilance decrement, which are based on extensively validated and intensively studied behavioral paradigms. Given the sensitivity of behavioral effects to characteristics of the vigilance task used, ERP effects would also be expected to differ between traditional and modified vigilance paradigms. Koelega and Verbaten (1991) reviewed early work using EEG to examine the vigilance decrement. A majority of those studies investigated auditory, or auditory and visual paradigms (Davis, 1964; Wilkinson et al., 1966; Wilkinson and Haines, 1970; Ford et al., 1976; Hink et al., 1978; Donald and Young, 1982). Few examined the visual modality alone (Haider et al., 1964; Harkins, 1974; Parasuraman and Davies, 1975; Parasuraman, 1985), and of those, many involved pharmacological manipulations (Strauss et al., 1984; Rohrbaugh et al., 1987). The amplitudes and latencies of ERP components, along with the very components that appear, vary somewhat by stimulus modality (Luck, 2014). In what follows, we focus on the visual modality, describing key ERP components identified in Koelega and Verbaten’s (1991) review, including the visual N1, the P2, and the P3b, in the time since.

The N1 is usually the first negative peak seen in an ERP waveform. It is usually presented as a negative deflection that occurs from 150 to 200 ms post-stimulus. It is elicited by visual stimuli and is associated with selective attention. The N1 peaks earliest over the anterior scalp and then over more posterior sites (Mangun and Hillyard, 1991). The lateral occipital N1 subcomponent (posterior subcomponent) has a larger amplitude in discrimination tasks as compared to detection tasks (Vogel and Luck, 2000), and is largest contralateral to a visual stimulus that appears at an attended location. Vigilance studies measuring the N1 have reported non-significant increases in amplitude and decreases in latency for non-critical stimuli (Haider et al., 1964). More recently, Boksem et al. (2005) found that N1 amplitude for both critical and non-critical stimuli decreased across blocks. In terms of N1 latency, most studies have reported no change or a non-significant increase in latency with time-on-task (Haider et al., 1964; Parasuraman and Davies, 1975; Koelega et al., 1992).

The P2 follows the N1 wave (at 200–300 ms) in the ERP waveform, has larger amplitude for critical event relevant features, and is enhanced when the critical stimulus has simple features (Luck, 2014). It appears over the central scalp in auditory, visual, and somatosensory tasks (Crowley and Colrain, 2004). The P2 is also enhanced when critical events are infrequent (Luck and Hillyard, 1994). P2 latency has been shown to increase with time-on-task for non-critical events in easy and difficult vigilance tasks (Harkins, 1974). Additionally, P2 amplitude has been found to increase to non-critical stimuli as behavioral performance declines with time-on-task (Harkins, 1974).

The P3b (P300) is a positive wave that peaks 300 ms or later after stimulus onset and is greatest over the parietal scalp (Patel and Azzam, 2005; Luck, 2014). The P3b is traditionally studied using the oddball paradigm, and is thought to involve stimulus evaluation/categorization (Patel and Azzam, 2005). The P3b is maximal following the appearance of low-probability critical stimuli. Vigilance studies measuring the P3b have consistently shown that its amplitude decreases with time-on-task (Koelega et al., 1992; Mockel et al., 2015). P3b latency, in turn, has been shown to increase with time-on-task for hits and false alarms (Parasuraman and Davies, 1975; Koelega et al., 1992).

In an effort to bridge the gap between the behavioral results and EEG/ERP activity, we replicated a well-established vigilance paradigm from Hitchcock et al. (1999) in the visual modality while continuously recording EEG. Based on past ERP studies of time-on-task effects, we expected to observe decreased amplitudes along with increased latencies of ERP components. Specifically, we expected N1 (posterior) amplitude to decrease and its latency to remain constant with time-on-task. We expected P2 amplitude to become more similar for critical and non-critical events with time-on-task, and for P2 latency to increase to non-critical events with time-on-task. For the P3b component, we expected to observe decreased amplitude and increased latency with time-on-task.

## Materials and Methods

### Subjects

Thirty-two participants (22 female, 10 male, 27 right handed, mean age 22.6 years, standard deviation 4.08, range 18–36) from the University of Dayton and surrounding community completed the study in a single 2-h session. All participants received monetary compensation. The data reported in this paper were part of a larger study that included three tasks completed in a fixed order: the Hitchcock Air Traffic Controller Task (40 min) (Hitchcock et al., 1999), the Psychomotor Vigilance Task (10 min; Dinges and Powell, 1985), and the 3-Stimulus Oddball Task (13 min) (Comerchero and Polich, 1999). In this paper, we focus on the Air Traffic Controller Task (for complete results from the other tasks, see Walsh et al., 2017).

The study was approved by the institutional review boards of the Air Force Research Laboratory and the University of Dayton. All participants gave written informed consent in accordance with the Declaration of Helsinki.

### Radar Task, Measurement, and Analysis

**FIGURE 1:**
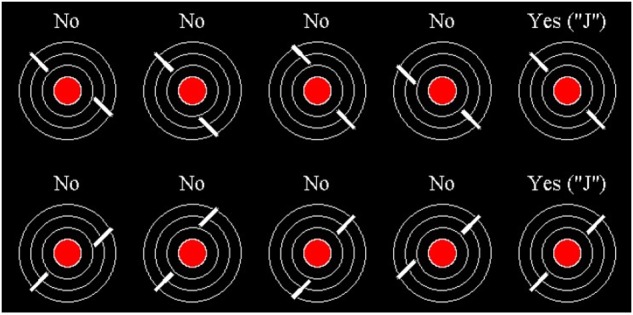
Radar task stimuli. All possible stimulus type variations. Stimuli with “No” above them are non-critical stimuli. Stimuli with “Yes (“J”)” above them are critical stimuli.

**Figure 1** shows the stimuli participants were instructed to attend to during the task. Participants were told that each stimulus represented a “city” with two “jets” approaching it. During the task, one stimulus appeared at the center of the computer screen at a time. Participants were instructed to identify when the two jets were on a collision course (i.e., the line segments were collinear) by pressing the “J” key on the keyboard with their right hand. Participants were instructed to withhold responses when the two jets were not on a collision course.

A single trial displayed one stimuli consisting of a red-filled central circle (“city”), measuring 1.6 cm in diameter and had three concentric rings (2.8, 3.9, and 5.1 cm) that were centrally aligned against a black background. Stimuli were presented with a Dell (64 bit, Intel Xeon 3.10 GHz processor, 16 GB RAM, Windows 7 OS) on a LCD computer monitor with a 60 Hz refresh rate approximately 60 cm away from the participant. The “jets” were two white line segments, each 0.15 cm wide by 1.4 cm long, superimposed on the concentric rings which corresponded to the jet flight paths. Stimulus displays appeared every 2 s and remained on screen for 300 ms. Critical events (i.e., collinear line segments) occurred in 3.3% of the trials. In all non-critical trials, line segments were offset from one another by 0.8 cm.

The task was presented via Psychophysics Toolbox, version 3.0.12 (Brainard, 1997) in MATLAB 2014b (MathWorks, Inc., Natick, MA, United States). A StimTracker Quad (Cedrus Corp., San Pedro, CA, United States) was used to record event codes synchronized to screen refreshes containing stimulus onset and offset. A USB2 keyboard was used to collect participants’ responses.

Participants were given 200 practice trials, during which they received feedback every 50 trials. Feedback consisted of the percentages of critical events detected and non-critical events ignored. Following a short break (less than 3 min), participants performed the task continuously for 1600 trials (40 min) without feedback or breaks.

### EEG Recording and Analysis

The EEG activity was captured on a separate Dell computer (64 bit, AMD Phenom 2.3 GHz processor, 2 GB RAM, Windows 7 OS) recording at the same time the experiment was running. A continuous EEG recording was acquired from 64 Ag-AgCl sintered electrodes using a BioSemi ActiveTwo (BioSemi, Amsterdam, Netherlands, Europe) system, using the 10–20 international electrode placement. Two flat, unlinked electrodes were applied to the left and right mastoids. Voltage offsets, an indicator of electrode-gel-skin connection quality, were reduced to less than the recommended ±40 mV (BioSemi, Amsterdam, Netherlands), and any problematic channels (drift, high frequency noise, etc.) were re-gelled and re-applied before beginning the recording. During the recording, scalp activity was re-referenced online to the linked CMS (common mode sense) and DRL (driven right leg) electrodes circuit. The recording was digitized at 512 Hz, and we applied digital low-pass (0.1 Hz) and high-pass (70 Hz) filters. Before analysis, the completed recordings were algebraically re-referenced to the average of the mastoids.

Electroencephalography signals were subjected to independent components analysis in EEGLAB (Delorme and Makeig, 2004) using the FastICA algorithm from Nolan et al. (2010). Components containing eye-blinks were identified and removed from the recording. Stimulus-locked epochs of 1200 ms were extracted from the continuous recording (including a 200 ms baseline), and were corrected over the pre-stimulus interval. Any epochs with voltages of ±100 mV were identified and removed. We binned epochs into two 20 min blocks to assess changes in scalp activity with time-on-task.

We analyzed ERP waveforms during four time windows: the N1 between 100 and 200 ms, the P2 between 200 and 300 ms, and the P3b between 300 and 450 ms and between 450 and 700 ms. We examined data from nine electrodes (F3, FZ, F4, C3, CZ, C4, P3, PZ, and P4), which together comprise three levels for the factor of frontoparietal location (frontal, central, and parietal) and three levels for the factor of laterality (left, midline, right).

## Results

### Behavioral Results

After excluding practice trials, we analyzed the effects of time-on-task on behavioral performance. To do so, we divided data from the 40-min experiment into four blocks, each lasting 10 min. In line with past vigilance studies, we examined how correct response time, *d′* (d-prime), and criterion varied with time-on-task (**Table 1**). Correct response time consisted of the response times recorded for trials where participants were supposed to hit the “J” key for critical stimuli and where they were supposed to refrain from pressing any key for the non-critical stimuli. Anything that was “missed”/incorrect was not counted in this response time. The sensitivity measure *d′* is a parametric measure calculated based on the difference between hits and false alarms. Criterion is a measure of a participants’ willingness to respond, or bias, independent of *d′*.

**Table 1:** Behavioral measures across experiment blocks with standard error in parentheses.

	Block 1	Block 2	Block 3	Block 4
Correct response time	637 (14)	677 (15)	675 (12)	686 (14)
*d*′	3.88 (0.25)	3.50 (0.23)	3.13 (0.20)	3.22 (0.22)
Criterion	0.22 (0.09)	0.29 (0.09)	0.48 (0.09)	0.43 (0.10)

#### Correct Response Time

Correct response time significantly increased from Blocks 1 to 2, with non-significant increases in the following blocks. We performed a one-way repeated measures analysis of variance (ANOVA) that included a single factor for experiment block. The main effect of block was significant, *F*(3,27) = 6.019, *p* < 0.001, ms = 15811.599, η^2^ = 0.712. Pairwise comparisons revealed that correct response times increased from Blocks 1 to 2, *t*(27) = 4.05, *p* < 0.001, *d* = 0.764, but not from Blocks 2 to 3 or from Blocks 3 to 4 (all *p* > 0.3).

#### Sensitivity (*d′*)

Sensitivity, assessed using *d′*, showed significant differences across the experiment, as confirmed using a one-way ANOVA, *F*(3,27) = 8.866, *p* < 0.001, ms = 3.866, η^2^ = 0.782. Pairwise comparisons revealed that *d′* decreased from Blocks 1 to 2, *t*(27) = 2.21, *p* < 0.05, *d* = 0.418, and from Blocks 2 to 3, *t*(27) = 3.10, *p* < 0.01, *d* = 0.587. Sensitivity did not decrease further after Block 3 *t*(27) = 0.67, n.s., *d* = 0.127.

#### Criterion

Criterion also showed significant differences across the experiment, as confirmed using a one-way ANOVA, *F*(3,27) = 4.398, *p* < 0.01, ms = 0.721, η^2^ = 0.716. Criterion did not change from Blocks 1 to 2, *t*(27) = 0.89, *p* > 0.1, *d* = 0.170, it increased from Blocks 2 to 3, *t*(27) = 2.88, *p* < 0.01, *d* = 0.545, and it did not change further after Block 3 *t*(27) = 0.62, n.s., *d* = 0.117.

To facilitate comparison with the ERP results, which were generated by experiment half, we also compared behavioral performance during the first and the second halves of the experiment. Correct response times increased from 655 to 681 ms, *t*(27) = 2.514, *p* < 0.05, *d* = 0.475. Sensitivity, assessed using *d*′, decreased from 3.62 to 3.16, *t*(27) = 3.150, *p* < 0.01, *d* = 0.595. Lastly, Criterion increased from 0.28 to 0.47, *t*(27) = 2.650, *p* < 0.05, *d* = 0.500.

### ERP Results

We first analyzed the ERP data after averaging waveforms across the entire experiment to establish the components of interest. There was not enough statistical power from trials for the EEG data to split the ERP data into four blocks like the behavioral results for comparison. Instead, we analyzed data by experiment half, combining across Blocks 1 and 2, and Blocks 3 and 4. At the end of this section, we also include a comparison of the behavioral data by experiment half to the ERP results to determine a relationship between the two.

#### Complete Experiment

**FIGURE 2:**
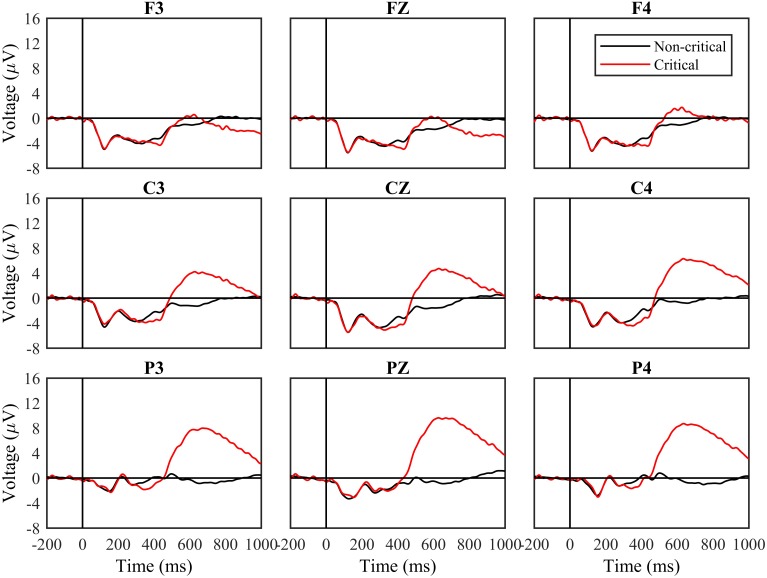
Event-related potential (ERP) waveforms by stimuli type for the entire task. Waveforms following critical and non-critical stimuli. Red line indicates critical stimuli ERP waveforms and the black line indicates non-critical ERP waveforms. The “0” time point represents when the stimulus appeared.

**Figure 2** shows ERPs from nine sites based on stimulus type (Critical and Non-Critical), with data aggregated across all experiment blocks. We analyzed data from four time windows of interest using a series of 2 (stimulus type) × 3 (frontoparietal location) × 3 (laterality) repeated measures ANOVAs. Complete results from all ANOVAs are contained in **Table 2**.

**Table 2:** Results from ANOVAs performed over four time windows.

	Time window			
	100–200 ms	200–300 ms	300–450 ms	450–700 ms
Stimulus type: *F*(1,27)	0.03	0.37	1.47	19.38^∗∗∗^
Frontoparietal: *F*(2,54)	17.68^∗∗∗^	17.72^∗∗∗^	17.20^∗∗∗^	20.13^∗∗∗^
Laterality: *F*(2,54)	15.45^∗∗∗^	7.32^∗∗^	4.73^∗^	5.35^∗∗^
Stimulus type × Frontoparietal: *F*(4,108)	1.01	0.20	1.42	39.61^∗∗∗^
Stimulus type × Laterality: *F*(4,108)	0.73	0.44	1.61	8.11^∗∗^
Frontoparietal × Laterality: *F*(4,108)	5.02^∗∗^	4.90^∗∗^	1.65	6.52^∗∗∗^
Stimulus type × Frontoparietal × Laterality: *F*(4,108)	1.37	1.25	3.82^∗∗^	4.67^∗∗^

^∗^*p < 0.05, ^∗∗^*p* < 0.01, ^∗∗∗^*p* < 0.001*.

From 100 to 200 ms, and from 200 to 300 ms, the periods of time containing the N1 and P2, the main effect of stimulus type and all interactions involving stimulus type were not significant (all *p* > 0.1; **Table 2**). From 300 to 450 ms, the main effect of stimulus type was not significant, *F*(1,30) = 1.47, n.s., but the three-way interaction between stimulus type, frontoparietal location, and laterality was significant, *F*(4,120) = 3.82, *p* < 0.01, *mse* = 1.336, η^2^ = 0.124. Voltages were more negative for critical vs. non-critical stimuli over the central scalp and at lateralized locations.

From 450 to 700 ms, a later period of time corresponding to the P3b, the main effect of stimulus type was significant, *F*(1,30) = 19.38, *p* < 0.0001, *mse* = 1713.052, η^2^ = 0.418. The two-way interactions between stimulus type and frontoparietal location [*F*(2,60) = 39.61, *p* < 0.001, *mse* = 472.342, η^2^ = 0.595] and laterality [*F*(2,60) = 8.11, *p* < 0.001, *mse* = 12.362, η^2^ = 0.231] were both significant, as was the three-way interaction, *F*(4,120) = 4.67, *p* < 0.01, *mse* = 3.942, η^2^ = 0.148. Voltages were more positive for critical vs. non-critical stimuli across the scalp, and the difference was greatest over parietal sites.

#### Experiment Halves

**FIGURE 3:**
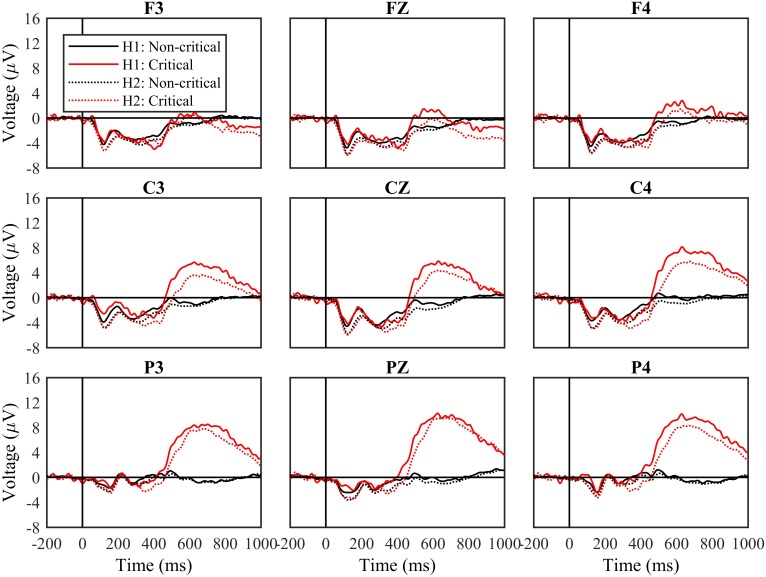
Waveforms by stimulus type and experimental half. Waveforms following non-critical and critical stimuli, and from the first (H1) and second (H2) halves of the experiment. Black solid lines indicate non-critical stimuli and red solid lines indicate critical stimuli from the first half the experiment. Black dotted lines show the non-critical stimuli and the red dotted lines show the critical stimuli in the second half of the experiment. The “0” time point represents when the stimulus appeared.

Next, we divided data between blocks that comprised the first half of the experiment (Blocks 1 and 2) and the second half of the experiment (Blocks 3 and 4). **Figure 3** shows ERPs based on stimulus type separately for the first and second halves of the experiment. We analyzed data from the time windows of interest in the same manner as before, but with the additional factor of experiment half.

From 100 to 200 ms, the main effect of experiment half was significant, *F*(1,27) = 18.42, *p* < 0.001, *mse* = 390.127, η^2^ = 0.411, while the effect of stimulus type was not, *F*(1,27) = 0.27, n.s., *mse* = 4.075, η^2^ = 0.010. The interaction between experiment half and stimulus type was also not significant, *F*(1,27) = 2.95, n.s., *mse* = 21.583, η^2^ = 0.078. For both types of stimuli, waveforms were more negative from 100 to 200 ms during the second half of the experiment.

From 200 to 300 ms, the main effects of experiment half and stimulus type were not significant, and nor was their interaction.

From 300 to 450 ms, the effect of experiment half was significant, *F*(1,27) = 6.49, *p* < 0.05, *mse* = 202.522, η^2^ = 0.194, and the effect of stimulus type was not, *F*(1,27) = 1.22, n.s., *mse* = 77.549, η^2^ = 0.043. For both types of stimuli, waveforms were more positive from 300 to 450 ms during the second half of the experiment. The interaction between experiment half and stimulus type was not significant, *F*(1,27) = 0.16, n.s., *mse* = 2.607, η^2^ = 0.006, but the three-way interaction between experiment half, stimulus type, and frontoparietal location was, *F*(2,54) = 3.50, *p* < 0.05, *mse* = 6.255, η^2^ = 0.115. Waveforms were more negative following critical vs. non-critical stimuli over central and parietal sites during the first half of the experiment, but not during the second half.

Finally, from 400 to 750 ms, the effect of experiment half was significant, *F*(1,27) = 4.66, *p* < 0.05, *mse* = 266.486, η^2^ = 0.147, as was the effect of stimulus type, *F*(1,27) = 18.58, *p* < 0.001, *mse* = 3837.830, η^2^ = 0.408. Waveforms were more positive from 400 to 750 ms during the first half of the experiment. The interaction between experiment half and stimulus type was not significant, *F*(1,27) = 1.54, *n.s.*, *mse* = 61.525, η^2^ = 0.054, reflecting the fact that waveforms remained more positive for critical vs. non-critical stimuli across the complete experiment.

In addition to examining the amplitude of components, we examined their latencies, quantified at the times when they reached peak amplitudes within the predefined windows. The latency of the P3b was of particular interest. We measured P3b peak latency from 400 to 750 ms at site Cz, and found that it increased from 594 to 625 ms over the course of the experiment, *t*(27) = 3.45, *p* < 0.01, *d* = 0.653.

#### Relationships Between Brain Signals and Behavioral Results

Did changes in neural signals from the first to the second half of the experiment relate to changes in behavior at the level of individual participants? In particular, based on the idea that the P3b relates to stimulus categorization, one might expect for changes in its latency to relate to changes in the time to respond to critical signals. Likewise, changes in the amplitude of the P3b might relate to changes in classification accuracy, as measured using *d*′.

To test these hypotheses, we calculated the differences in correct response times and *d*′ between the first and the second half of the experiment for all participants, and we calculated the differences in P3b latency and P3b amplitude. Changes in response latency were positively associated with changes in P3b latency, though not reliably so (*r* = 0.16, n.s.). Changes in *d*′ were also positively associated with changes in P3b amplitude, *r* = 0.41, *p* < 0.05. Participants who showed the greatest decrease in *d*′ from the first to the second half of the experiment also showed the greatest decrease in P3b amplitude to critical events.

## Discussion

The goal of this study was to examine the neural basis of the vigilance decrement using an extensively validated behavioral paradigm. Our study yielded four clear results. First, behavioral performance, as assessed using *d*′ and correct response time, declined over the course of the experiment, consistent with past research on the vigilance decrement. Second, the amplitude of the N1 to critical and non-critical signals alike increased with time-on-task. Third, the amplitude of the P3b decreased over the course of the experiment. The change in P3b amplitude also related to individual differences in the size of the vigilance decrement, as assessed using *d*′. Fourth and finally, the latency of the P3b increased with time-on-task. We consider the implications of each of these findings with respect to the vigilance decrement and corresponding theoretical accounts in turn.

We observed decreased behavioral sensitivity, assessed using *d*′, from the first to the second half of the experiment. This was accompanied by an increase in correct response times to critical signals. These findings corroborate past research. Mackworth (1948) first assessed that the accuracy of signal detections declined by 10–15% after about 30 min, and continued to gradually decline after that. The meta-analysis performed by See et al. (1995, p. 242) also provided evidence for a “pervasive and sizeable” vigilance decrement in validated paradigms. Commensurate with these earlier findings, we found a delay in correct response times across the first 20 min of the experiment before reaching an asymptote, and that sensitivity decreased over the first 30 min of the experiment before stabilizing.

The ERP results of our experiment were multifaceted, and partially predicted by past studies of time-on-task effects using non-vigilance paradigms. The amplitude of the N1 for critical and non-critical stimuli increased from the first to the second half of the experiment. This is partially consistent with a past study by Haider et al. (1964) that reported increased N1 amplitude to non-critical stimuli with time-on-tasks. The N1 is associated with selective attention, and its amplitude is enhanced when items appear at attended locations. One interpretation of this result, then, is that the increased amplitude of the N1 with time-on-task was driven by increased compensatory effort by participants to remain attentive for stimulus onset. In a related study, Wang et al. (2016) used a 160 min cued Stroop task to investigate compensatory brain activity in response to cognitive fatigue. They found that the amplitude of the anterior frontal ERP increased during their compensatory period as time-on-task increased.

Boksem et al. (2005) found the opposite effect, decreased N1 amplitude with time-on-task. This inconsistency could relate to either of two factors. First, Boksem et al. (2005) studied a different N1 component over the lateral-occipital scalp region, whereas we studied the N1 over the frontal and central scalp. Second, Boksem et al. (2005) used an entirely different paradigm that required comparison of a presented letter to a set of letters held in memory. These measurements and methodological distinctions prevent direct comparison, but do raise issues that will be important to address in future research.

The amplitude of the P3b decreased from the first to the second half of the experiment, consistent with earlier studies that examined time-on-task effects (Koelega et al., 1992; Hopstaken et al., 2015; Mockel et al., 2015). Changes in P3b amplitude related to individual differences in the size of the vigilance decrement. The triarchic model of P3b (Johnson, 1986) relates its amplitude to three factors: stimulus probability, effort, and task difficulty. Stimulus probability remained constant across our experiment. As such, the decreased amplitude of the P3b related to one of the remaining factors: decreased effort or increased task difficulty. In light of the finding that the amplitude of N1 increased, suggesting increased effort, we are inclined to adopt the final account – that the reduced amplitude of P3b related to increased difficulty with time-on-task. Paralleling the ERP findings in this study, behavioral response sensitivity decreased over time.

Lastly, we found that the latency of the P3b increased with time-on-task. The emerging consensus is that the P3b relates to contextual updating (Luck, 2014). When a stimulus deviates from an expected standard held in memory, the representation is updated producing an enhanced P3b (McCarthy and Donchin, 1981; Polich, 2007; but see Verleger, 1988). Implied in this, and other accounts is that the P3b only appears once a stimulus has been categorized. This would suggest, in the context of our vigilance paradigm, that classification became increasingly delayed with time-on-task, accounting for the corresponding increase in response times. Past studies have also found increased P3b latency with time-on-task (Parasuraman and Davies, 1975; Koelega et al., 1992), again suggesting that the effects of time-on-task, as reflected in the vigilance decrement, manifest prior to the completion of stimulus classification.

To reiterate, the results provide important evidence regarding the nature of the vigilance decrement, and also inform the ongoing debates about the underlying mechanisms responsible for the observed performance decrement. Specifically, the increased amplitude of the N1 and the decreased amplitude of the P3b from the first half of the experiment to the second suggest that the ability to sustain attention decreased over time. This finding is consistent with overload accounts, in which information processing resources are depleted over time, increasing task difficulty and leading to the vigilance decrement. However, the increase in N1 amplitude in the second half of the study contrasts previous findings. Perspective is needed when examining these results within the context of past and future research. It is difficult to compare the overload and underload accounts of the vigilance decrement because there is a skewed amount of behavioral research performed in favor of the overload accounts. Both theories also utilize “different” vigilance paradigms, meaning that the overload account has participants respond to infrequent critical stimuli, while the underload account has participants respond to frequent non-critical stimuli. While this may arguably be comparable behaviorally, ERP waveforms can change with such subtlety. It would be beneficial to uncover how each paradigm affects the ERP waveform to make informed interpretations of how the underlying neural activity fits the observable behavioral phenomenon.

The interpretation of the results is complicated in some cases. The P3b results may support overload accounts when considered in conjunction with the decreasing amplitude of the N1 from the first half of the experiment to the second. However, the decrease in the P3b in the second half of the experiment could also reflect decreased effort, workload, or task engagement, which may provide support to underload accounts. Likewise, the decrease in the P3b amplitude could also provide support for the resource-control theory, depending on how the theory itself is conceptualized. For example, withdrawal of executive control could impact stimulus processing and categorization in a manner that would decrease the amplitude of the P3b and delay its peak. Yet the finding of an enhanced N1 seems to argue against both the underload account and the resource-control theory, to the extent that it reflects increased effort to remain attentive to stimulus processing.

Future studies are needed to differentiate between these alternative interpretations of the vigilance decrement. In the introduction, we identified a number of factors that influence the magnitude of the observed behavioral deficits. Systematic investigation of the changes in neural activity that accompany these behavioral changes will provide critical information about the nature of the mechanisms underlying the vigilance decrement. The research presented here starts to establish the empirical foundation that is needed to inform our understanding of such issues. We have identified several changes in ERP patterns associated with the emergence of the vigilance decrement in a well-validated vigilance task. The behavioral results are in line with previous investigations, and the ERP data provide novel results and insight into the underlying neuro-functional changes that are associated with those performance deficits. It is also important to consider that there are limits to neurological studies, specifically with EEG. For instance, using EEG with traditional vigilance studies necessitates long collection periods to have enough statistical power, and data analyses can be complex. There can also be issues of sensory and motor, and trial confounds to look for in ERP waveforms or when considering study design. Careful study design utilizing EEG experiments and future research is needed to characterize the changes in our study in greater detail, and to inform ongoing theoretical debates about the nature of the vigilance decrement.

## Ethics Statement

This project was approved by the Air Force Research Laboratory Institutional Review Board. Prior to data collection at the University of Dayton Research Institute, the University of Dayton Institutional Review Board reviewed the approved documents from the Air Force and conferred approval before any human subject data was collected. All subjects gave written informed consent prior to participating in the study. They were all notified of ability to ask questions before, during, and after the study, as well as the voluntariness of their participation.

## Author Contributions

The authors (AH, MaW, RB, MM, MeW, MK, and GG) confirm substantial contributions to the conception or design of the work, or the acquisition, analysis, and interpretation of data for the work. They also confirmed assistance with drafting and critically revising the intellectual content and approval of the final version to be published. All authors agree to be held accountable for all aspects of the work to ensure that questions related to the accuracy or integrity of any part of the work are appropriately investigated and resolved.

## Conflict of Interest Statement

MaW is employed by the RAND Corporation. MM is employed by Ball Aerospace. MK is employed by L3 Technologies. The other authors declare that the research was conducted in the absence of any commercial or financial relationships that could be construed as a potential conflict of interest. The reviewer GB and handling Editor declared their shared affiliation.
